# Carbenoxolone Treatment Ameliorated Metabolic Syndrome in WNIN/Ob Obese Rats, but Induced Severe Fat Loss and Glucose Intolerance in Lean Rats

**DOI:** 10.1371/journal.pone.0050216

**Published:** 2012-12-17

**Authors:** Siva Sankara Vara Prasad Sakamuri, Mahesh Sukapaka, Vijay Kumar Prathipati, Harishankar Nemani, Uday Kumar Putcha, Shailaja Pothana, Swarupa Rani Koppala, Lakshmi Raj Kumar Ponday, Vani Acharya, Giridharan Nappan Veetill, Vajreswari Ayyalasomayajula

**Affiliations:** 1 Department of Biochemistry, National Institute of Nutrition, Indian Council of Medical Research, Jamai Osmania PO, Hyderabad, Andhra Pradesh, India; 2 Department of Animal Physiology, National Centre for Laboratory Animal Sciences, Indian Council of Medical Research, Jamai Osmania PO, Hyderabad, Andhra Pradesh, India; 3 Department of Pathology, National Institute of Nutrition, Indian Council of Medical Research, Jamai Osmania PO, Hyderabd, Andhra Pradesh, India; Catholic University Medical School, Italy

## Abstract

**Background:**

11beta-hydroxysteroid dehydrogenase type 1 (11β-HSD1) regulates local glucocorticoid action in tissues by catalysing conversion of inactive glucocorticoids to active glucocorticoids. 11β-HSD1 inhibition ameliorates obesity and associated co-morbidities. Here, we tested the effect of 11β-HSD inhibitor, carbenoxolone (CBX) on obesity and associated comorbidities in obese rats of WNIN/Ob strain, a new animal model for genetic obesity.

**Methodology/Principal Findings:**

Subcutaneous injection of CBX (50 mg/kg body weight) or volume-matched vehicle was given once daily for four weeks to three month-old WNIN/Ob lean and obese rats (n = 6 for each phenotype and for each treatment). Body composition, plasma lipids and hormones were assayed. Hepatic steatosis, adipose tissue morphology, inflammation and fibrosis were also studied. Insulin resistance and glucose intolerance were determined along with tissue glycogen content. Gene expressions were determined in liver and adipose tissue. CBX significantly inhibited 11β-HSD1 activity in liver and adipose tissue of WNIN/Ob lean and obese rats. CBX significantly decreased body fat percentage, hypertriglyceridemia, hypercholesterolemia, insulin resistance in obese rats. CBX ameliorated hepatic steatosis, adipocyte hypertrophy, adipose tissue inflammation and fibrosis in obese rats. Tissue glycogen content was significantly decreased by CBX in liver and adipose tissue of obese rats. Severe fat loss and glucose- intolerance were observed in lean rats after CBX treatment.

**Conclusions/Significance:**

We conclude that 11β-HSD1 inhibition by CBX decreases obesity and associated co-morbidities in WNIN/Ob obese rats. Our study supports the hypothesis that inhibition of 11β-HSD1 is a key strategy to treat metabolic syndrome. Severe fat loss and glucose -intolerance by CBX treatment in lean rats suggest that chronic 11β-HSD1 inhibition may lead to insulin resistance in normal conditions.

## Introduction

Glucocorticoids are essential for the normal functioning of immune, cardiovascular and central nervous systems in the body. They regulate cellular metabolism, differentiation and development. Glucocorticoids are also implicated in the pathogenesis of chronic diseases like obesity, metabolic syndrome and type 2 diabetes. In Cushing's syndrome patients, elevated circulatory glucocorticoids are associated with all major features of metabolic syndrome like visceral obesity, hypertriglyceridemia, hypercholesterolemia and insulin resistance. However, the role of plasma glucocorticoids in the development of idiopathic obesity is not clear, as their levels are not altered or even low in obese patients [1].

Glucocorticoid-sensitivity of target tissues depends on plasma hormone level, density of glucocorticoid receptors and local metabolism by 11β-hydroxysteroid dehydrogenases (11β-HSDs). 11β-hydroxysteroid dehydrogenase type 1 (11β-HSD1) converts inactive glucocorticoids (cortisone in humans and 11-dehydrocorticosterone in rodents) to active glucocorticoids (cortisol in humans and corticosterone in rodents) and thereby increases local glucocorticoid action. It is expressed in various tissues including liver, adipose tissue and brain [2]. 11β-HSD2 catalyses the reverse reaction and expressed in distal nephron, sweat and salivary glands [3].

Recent studies showed that 11β-HSD1 plays an important role in the development of obesity and associated co-morbidities. In animal models of obesity and human obesity, 11β-HSD1 activity is elevated in adipose tissue [4]–[6]. Adipocyte-specific overexpression of 11β-HSD1 in mice resulted in the development of visceral obesity, dyslipidemia, and insulin resistance [7], whereas 11β-HSD1 knock-out mice are resistant to diet-induced obesity and have displayed improved insulin sensitivity [8]. Liver-specific over-expression of 11β-HSD1 in mice results in the elevation of plasma triglycerides and insulin resistance [9]. Carbenoxolone (CBX), a non-selective inhibitor of 11β-HSD1 (inhibits 11β-HSD2 also) is shown to reduce fat mass, plasma triglyceride and cholesterol levels in obese rodent models [10], [11]. CBX is also shown to improve insulin sensitivity in animal models and humans [11], [12]. Recently, a variety of selective 11β-HSD1 inhibitors are developed and shown to ameliorate metabolic syndrome features in animal models of obesity and diabetes [13]–[16].

WNIN/Ob obese rat strain is developed from 80 year-old, inbred wistar rat colony at National Centre for Laboratory Animal Sciences (Hyderabad, India) [17]. They exhibit metabolic syndrome features like visceral obesity, hypertriglyceridemia, hypercholesteremia and hyperinsulinaemia [17]. WNIN/Ob obese rat is monogenic mutant and preliminary studies on WNIN/Ob obese rats show no molecular defects in leptin and leptin receptor [unpublished data]. Positional cloning studies confirmed the presence of mutation on 5th chromosome and gene sequencing studies are in progress to characterize the mutation [unpublished data]. Our previous studies have shown elevated 11β-HSD1 activity in adipose tissue of WNIN/Ob obese rats, which is similar to the observations in human and other animal models of obesity [18]. Novel feature of this model with respect to 11β-HSD1 activity is that, higher enzyme activity in adipose tissue is observed during fasting state but not in fed state, wherein activity is low [18].

As 11β-HSD1 inhibition is known to decrease obesity and ameliorate co-morbidities like dyslipidemia and insulin resistance in animal and human obesity, here, we are interested to study the effect of 11β-HSD1 inhibition on these parameters in WNIN/Ob obese rat. For this, we injected CBX (50 mg/Kg body weight) subcutaneously to WNIN/Ob lean and obese rats for four weeks and studied its effects on body composition, plasma lipids and insulin resistance. Here, we are also reporting the effect of 11β-HSD1 inhibition on adipose tissue inflammation, fibrosis and tissue glycogen content in liver and adipose tissue in obese condition. Further, to explain the observed changes in metabolic syndrome-parameters, we have studied the expression of genes involved in lipid metabolism.

## Materials and Methods

### Animal experiment

Animal experiment and protocols were approved by Institutional Animal Ethical Committee of National Centre for Laboratory Animal Sciences (NCLAS), Hyderabad, India (IAEC code no. P25/8-2009/SS). Three month-old, male, 12 lean and 12 obese rats of WNIN/Ob strain (National Centre for Laboratory Animal Sciences (NCLAS), Hyderabad, India) were divided into two subgroups consisting of 6 rats each. Animals were maintained under controlled conditions of light (12 h day and 12 h night) and temperature (21°C) and allowed free access to standard pellet diet (NCLAS, Hyderabad, India) and drinking water. After one week of acclimatization, drug treatment was commenced. Subcutaneous injection of CBX (50 mg/kg body weight) was given to lean and obese rats of experimental group daily between 9.00AM to10.00AM for four weeks. The concentration of CBX solution was 0.1 mg/ml (autoclaved distilled water was used as solvent). Volume-matched distilled water (vehicle) was given to lean and obese rats of control group. Bodyweights were recorded weekly for accurate dosing of drug and to follow the progress of weight gain. Food intake was measured twice a week for each animal. At the end of four weeks of treatment, exactly one hour after the carbenoxolone injection, animals were sacrificed by CO_2_ asphyxiation. Tissues were dissected out, snap-frozen in liquid nitrogen and stored at −80°C until the analysis.

### Oral glucose- tolerance test (OGTT)

OGTT was performed at the end of the third week. After overnight fasting, glucose (200 g/l) was administered oro-gastrically at a dose of 2.0 g/kg body weight and blood samples were collected from supra-orbital sinus at 0,30,60 and 120 min. Glucose and insulin levels were measured at all time points. Fasting blood at zero time point was used for analysis of plasma triglycerides, cholesterol, HDL-cholesterol and corticosterone.

### Insulin resistance and glucose- tolerance

Insulin resistance was assessed from homeostasis model assessment of insulin resistance (HOMA-IR) and was calculated from fasting glucose and insulin values using the following formula.

### HOMA-IR = (fasting insulin [µU/ml] × fasting glucose [mmol/l])/22.5

Area under curve (AUC) for insulin and glucose was calculated during OGTT by the trapezoidal method [19]. Glucose- tolerance was assessed by calculating glucose AUC.

### Body composition

Body composition of experimental animals was assessed at the end of experiment by Total Body Electrical Conductivity (TOBEC) using small animal body composition analysis system (EM-SCAN, Model SA-3000 Multi detector, Springfield, USA). Lean body mass, fat-free mass and total body fat percent were calculated as described previously [20].

### Plasma parameters

Plasma insulin levels were measured by radioimmunoassay (RIA) kit (BARC, India). Plasma corticosterone levels were measured by RIA kit (Siemens, Los Angeles, USA). Plasma glucose, triglycerides, total cholesterol and HDL-cholesterol levels were measured by using commercially available enzyme-based assay kits (Biosystems, Barcelona, Spain).

### Tissue glycogen

Tissue glycogen levels in the liver and adipose tissue were determined as described previously [21]. Briefly, liver (50 mg) and retroperitoneal adipose tissue (200 mg) were homogenized in 0.03N HCl (20 µl/mg tissue) on ice. Aliquots of homogenate (200 µl) are mixed with equal volume of 2N HCl and incubated for 2 h at 90°C. Tissue homogenates are immediately neutralized with equal volume of 2N NaOH. Total glucose was estimated in the acid-hydrolyzed tissue homogenates. For free glucose estimation, homogenates were immediately neutralized with 2N NaOH, after addition of 2N HCl without the boiling step. Total glycogen was estimated by subtracting the free glucose values from total glucose values. Glucose was estimated by using commercial kits (Biosystems, Barcelona, Spain).

### Liver triglycerides

Lipid fraction from liver was extracted by method of Folch et.al [22]. Briefly, tissue was homogenized in chloroform: methanol mixture (2∶1v/v) for 2–3 minutes (1 g in 20 mL of solvent). After homogenization, butylated hydroxytoluene (BHT) was added (0.1 mg %), mixed thoroughly, filtered and washed with 0.9% KCl solution. The mixture was allowed for phase separation and lower lipid phase was collected, evaporated under dry nitrogen and dissolved in iso-propanol. Triglycerides were estimated by commercially available kit (Biosystems, Barcelona, Spain).

### Histopathology

Liver and retroperitoneal adipose tissue samples were fixed in 10% neutral buffered formalin, embedded in paraffin and 4μ sections were taken for staining. Adipose tissue samples were stained with haematoxylin and eosin (H&E) to study adipocyte-hypertrophy (calculated by number of cells/16sq.mm) and also to assess the ‘crown like structures,’ which are the hallmark of adipose tissue inflammation. Liver sections were stained with H&E and oil red O (ORO) to study hepatic steatosis. Masson's trichrome (MT) staining was performed for both the tissues to observe fibrosis. Liver glycogen was detected by using periodic acid-schiff's stain (PAS). Images were taken with Nikon eclipse e800 microscope (Nikon Corporation, Tokyo, Japan) and analyzed with Image-Pro Plus software (Media cybernetics, Bethesda, USA). For histopathology of liver, samples from all the groups were used, but for adipose tissue, only samples from obese rats were processed (due to tissue limitation in lean rats).

### Gene expression in liver and adipose tissue by semi-quantitative reverse transcription PCR

Total RNA was isolated from liver and retroperitoneal adipose tissue, using Trizol RNA isolation kit (Invitrogen, Carlsbad, CA). 2 µg of RNA was used to synthesize first strand cDNA. The reverse transcription (RT) reaction was carried out by incubating RNA with 0.5 µg oligo- dT primer (sigma) and 100 units of Molony murine leukemia virus reverse transcriptase (Finnzymes, Espoo, Finland) at 37°C for 60 min.Total reaction volume used in RT was 20 µL. An aliquot of cDNA was amplified in a 20 µL PCR reaction mixture. PCR conditions were as follows: denaturation at 94°C for 1 min, annealing at 60–64°C for 45 seconds and polymerization for 70°C for 1 min with DyNAzyme II DNA polymerase (Finnzymes, Espoo, Finland). A final extension was carried out at 70°C for 7 min. The amount of RNA and the annealing temperature for different genes were standardized for linearity. Sequences of primers used for amplification are Stearoyl CoA desaturase 1 (SCD1):forward primer (FP)-5′-CGGCCCACATGCTCCAAGAGATCT-3′ and reverse primer (RP)-5′–GTCTTCTTCCAGATAGAGGGGCACC-3′, Malic enzyme (ME1):FP-5′-ATAAAGTGACCAAGGGCCGTGCG-3′ and RP-5′-ACAGGCCACTACCCCAAGAGCAA-3′, Lysosomal lipase (LIPA):FP-5′-CGGTATCCAAAGAGACGGCTGCA-3′ and RP-5′-ACAGGCCTCGATAAATTAGGGCCT-3′, Macrophage-expressed gene 1 (MPEG1): FP-5′-TCTTGCTGGTGAATGCCTGGGAC-3′ and RP-5′-ATACCCGGGTCTCTGAGAGGCTTG-3′ , Beta-3 adrenergic receptor (β3-AR):FP-5′-ACTTTCGCGACGCCTTCCGT-3′ and RP-5′-AGCCATCAAACCTGTTGAGCGGT-3′, Calnexin: FP-5′- GCAGCGACCTATGATTGACAACC-3′ and RP-5′-GCTCCAAACCAATAGCACTGAAAG-3′. Cholesterol 7α-hydroxylase (CYP7α): FP-5′-GTGGAGCTTTACAGAGTGCTGGCC-3′ and RP-5′-ATGCTGTCTAGTACCGGCAGGTCA-3′, Peroxisome proliferator-activated receptor-gamma coactivator 1α (PGC1α) : FP-5′-CGATGACCCTCCTCACACCA-3′ and RP-5′-TTGGCTTGAGCATGTTGCG-3′, Scavenger receptor class B1 (SR-B1): FP-5′-GCGTCGGGCAAACAGGGAAGAT-3′ and RP-5′-GCACCAGCTGCGTGTAGAACGT-3′, glyceraldehyde-3-phospahte dehydrogenase (GAPDH): FP-5′-GGGACCTCGGCTGCCATAGACATA-3′ and RP-5′-ACTTTGTCACAAGAGAAGGCAGCCC-3′. Glyceraldehyde-3-phosphate dehydrogenase (GAPDH) was used as internal control for relative quantification of liver gene expression, where as calnexin was amplified as an internal control for relative quantification of adipose tissue gene expression. After amplification, 8 µL of reaction mixture was subjected to agarose gel electrophoresis (2%) in Tris-acetate EDTA buffer (pH 8.2). The ethidium bromide- stained bands were visualized by a UV-transilluminator and analyzed densitometrically, using Quantity One software program (Bio-Rad, version 4.4.0).

### Enzyme activity

11β-HSD1 functions as a reductase *in vivo*, reactivating corticosterone from inactive 11-dehydrocorticosterone. However, in tissue homogenates, dehydrogenase activity predominates, hence 11β-HSD1 activity was measured by conversion of corticosterone to 11-dehydrocorticosterone. Liver, adipose tissue and skeletal muscle were homogenized in Krebs-Ringer buffer (118 mM NaCl, 3.8 mM KCl, 1.19 mM KH2PO4, 2.54 mM CaCl2, 1.19 mM MgSO4, and 25 mM NaHCO3, pH 7.4). Post- nuclear fractions from liver, adipose tissue and skeletal muscle were prepared by centrifuging tissue homogenate at 1000 g for 20 min. 11β-HSD1 activity was measured in post-nuclear fractions of liver and omental adipose tissue by incubating in duplicates at 37°C, in Krebs-Ringer buffer containing 0.2% glucose, 1 mM NADP and 50 nM 1, 2, 6, 7-[^3^H_4_] corticosterone (Amersham, UK). Conditions were optimized to ensure first order kinetics, by adjusting protein concentrations for liver (40 µg/ml), adipose tissue (1 mg/ml) and skeletal muscle (1 mg/ml). After incubation (30 min for liver and 6 h for adipose tissue and skeletal muscle), steroids were extracted with ethyl acetate. Ethyl acetate was evaporated under dry nitrogen and steroids were resuspended in mobile phase (50% water, 30% acetonitrile and 20% methanol). Steroids were separated by HPLC, using reverse phase C18 column and radioactive counts from substrate and product were calculated by online scintillation counter (IN/US systems, UK). Enzyme activity was expressed as percentage of substrate conversion.

### Immunoblotting

Gastrocnemius muscles were homogenized in Tris buffer containing 250 mM sucrose, 10 mM Tris (pH 7.4), 1 mM EDTA, 1 mM DTT supplemented with cocktails having protease and phosphatise inhibitors. Post-nuclear fractions were collected and fractions having 50 µg protein was used for protein expression studies by ECL chemiluminescence method (Amersham, UK), using antibody against Protein kianse B (PKB/Akt), phosphorylated Akt (Ser ^473^), protein-tyrosine phosphatise 1B (PTP1B) and β-actin (Cell Signaling Technology, MA, USA). β-actin was used to normalize the protein expression.

### Statistical analysis

Data were subjected to one way ANOVA using SPSS (version 11.0), followed by post-hoc test of the least significance difference. For parameters, where heterogeneity of the variance was significant, differences between groups were tested for log- transformed data or with non-parametric kruskal Wallis test. n = 6 for all the measurements, except for the gene expression, where n = 4. All values are presented as means ± SEM. The differences were considered significant at p value<0.05.

## Results

### Effect of CBX on 11β-HSD1 activity

**Figure 1:**
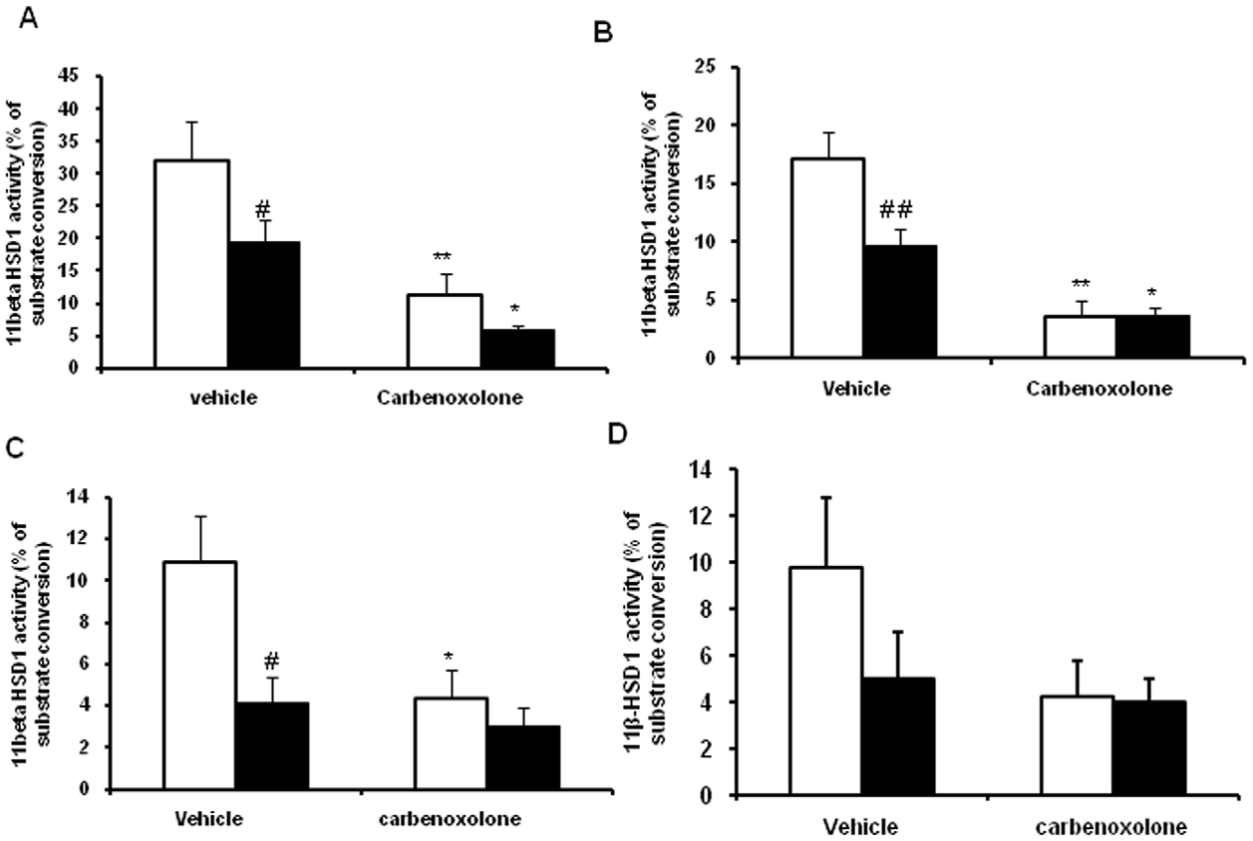
Effect of carbenoxolone on 11β-HSD1 activity in WNIN/Ob lean and obese rats. (A). Liver. (B). Subcutaneous adipose tissue. (C). Omental adipose tissue. (D). Skeletal muscle. 11β-HSD1 activity was measured after 1 h of subcutaneous injection of carbenoxolone or vehicle (50 mg/kg body weight/day). Empty bars indicate lean phenotype where as filled bars indicate obese phenotype. Values are means ± SEM for 6 animals for group. #*p*<0.05, ##*p*<0.01 and ### *p*<0.001 comparing vehicle-treated lean and obese rats. *p<0.05, **p<0.01 and *** *p*<0.001 comparing carbenoxolone-treated animals with vehicle-treated animals of the same phenotype.

Subcutaneous administration of CBX (50 mg/kg body weight) significantly decreased hepatic 11β-HSD1 activity by 65% in lean rats (p<0.001) and by 70% in obese rats (p<0.05) as compared with their respective vehicle-treated control rats (Figure 1). 11β-HSD1 activity in subcutaneous adipose tissue was significantly decreased by CBX in lean (79%, p<0.001) and obese rats (60%, p<0.05) as compared with their respective vehicle-treated control rats (Figure 1). CBX significantly decreased 11β-HSD1 activity in omental adipose tissue of lean rats (60%, p<0.01) but not in obese rats as compared with their control group rats (Figure 1). Skeletal muscle (Gastrocnemius) 11β-HSD1 activity showed a strong tendency towards decrease (p = 0.059) in both the phenotypes by CBX administration as compared with their respective control group rats (Figure 1).

### Effect of CBX on adrenal gland weight and plasma corticosterone

**Figure 2:**
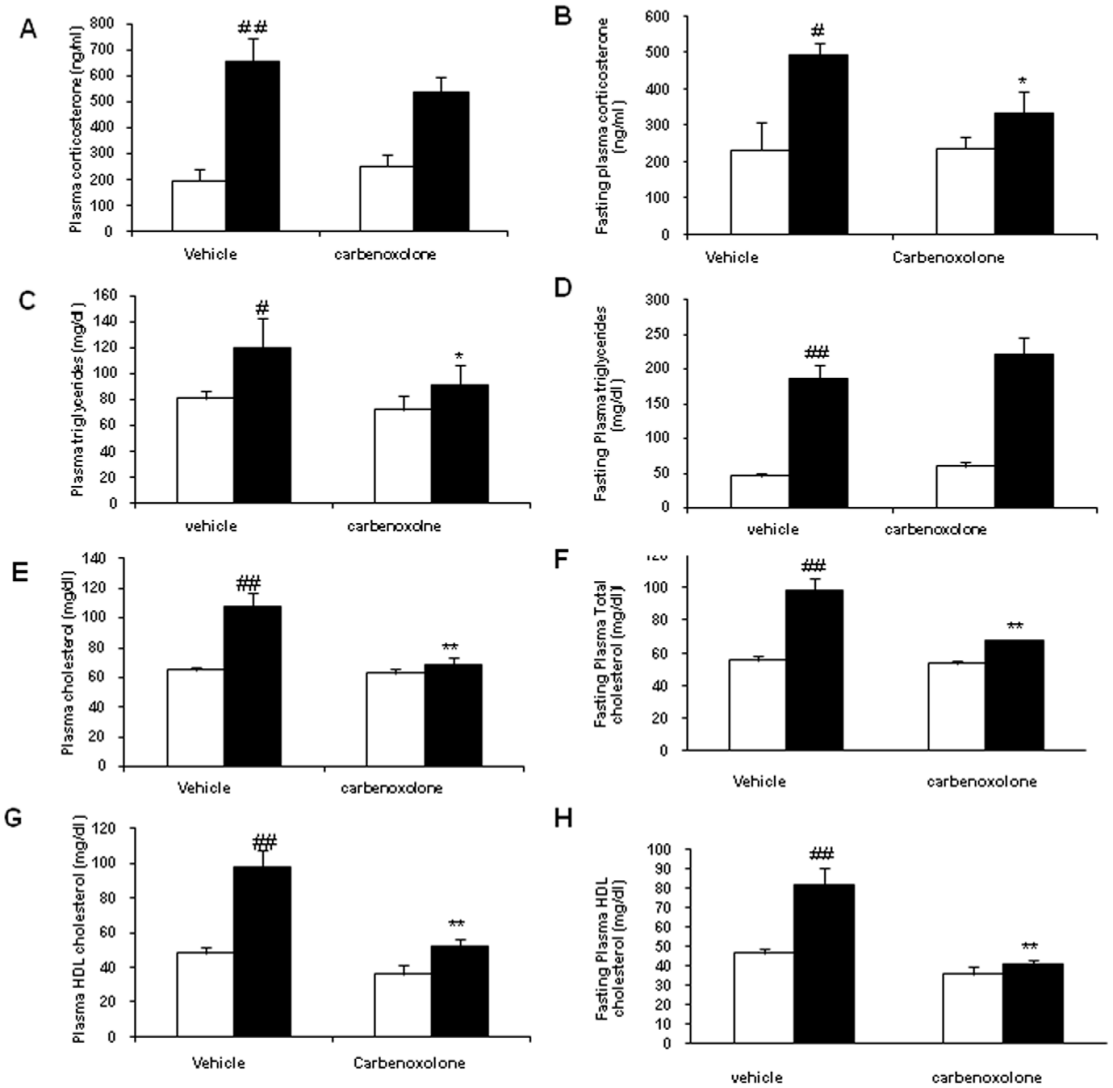
Effect of carbenoxolone on plasma biochemical parameters in WNIN/Ob lean and obese rats. (A). Fed-state corticosterone. (B). Fasting corticosterone. (C). Fed-state triglycerides. (D). Fasting triglycerides. (E). Fed-state total cholesterol. (F). Fasting total cholesterol. (G). Fed-state HDL cholesterol. (H). Fasting HDL cholesterol. Plasma parameters were measured after 4weeks of treatment with carbenoxolone or vehicle (50 mg/kg bodyweight/day). Empty bars indicate lean phenotype where as filled bars indicate obese phenotype. Values are means ± SEM for 6 animals for group. #*p*<0.05, ##*p*<0.01 and ### *p*<0.001 comparing vehicle-treated lean and obese rats. *p<0.05, **p<0.01 and *** *p*<0.001 comparing carbenoxolone-treated animals with vehicle-treated animals of the same phenotype.

Adrenal weight and plasma corticosterone levels were significantly higher in control obese rats as compared with their age and sex-matched control lean rats (Table 1 & Figure 2). Adrenal weights were not altered by CBX treatment in both the phenotypes (Table 1). CBX treatment significantly decreased fasting plasma corticosterone levels by 32.5% in obese rats (p<0.05) as compared with the vehicle-treated obese rats (Figure 2), which was not affected in lean rats (Figure 2). Fed-state corticosterone levels were not altered by CBX in both the phenotypes (Figure 2).

**Table 1:** Effect of carbenoxolone on food intake, body weight and organ weights in WNIN/Ob lean and obese rats.

Parameters	LC	OC	LT	OT
Pre-treatment body weight (g)	234.5±7.20	397.4±8.37**###**	230.8±5.68	396.0±9.63
Post-treatment body weight (g)	304.6±11.6	533.7±20.6##	280.8±6.8	508.3±17.2
Body weight gain (g)	70.2±6.16	136.3±17.50**###**	50.0±4.90*	112.3±18.70
Daily food intake (g)	18.1±0.31	30.4±1.50##	15.9±0.36**	27.1±1.67
Adrenal weight (mg)	35.5±1.17	45.1±3.31#	38.4±2.19	47.6±2.85
RPWAT (g)	1.9±0.12	17.9±1.22**###**	.92±0.30	19.7±1.90
OMWAT (g)	0.46±0.26	2.02±0.17**###**	0.24±0.07	1.09±0.20
EPIWAT (g)	2.2±0.17	13.3±0.90**###**	2.11±0.10	10.4±1.00
Gastrocnemius (g)	1.65±0.06	1.32±0.11#	1.48±0.18	1.26±0.10
Soleus (g)	0.33±0.02	0.27±0.03	0.30±0.02	0.26±0.02

Values represent means ± SEM of 6 rats per group. LC, Lean control; OC, Obese control; LT, Lean- treated; OT, Obese- treated. Parameters were measured after four weeks of treatment with carbenoxolone (50 mg/kg/body weight/day) or vehicle (same volume of phosphate buffer saline).

# *p*<0.05,

## *p*<0.01 and ### *p*<0.001 comparing vehicle-treated lean and obese rats.

* *p*<0.05,

** *p*<0.01 and *** *p*<0.001 comparing carbenoxolone- treated animals with vehicle-treated animals of the same phenotype.

### Effect of CBX on plasma triglycerides, total cholesterol and HDL-cholesterol

**Figure 3:**
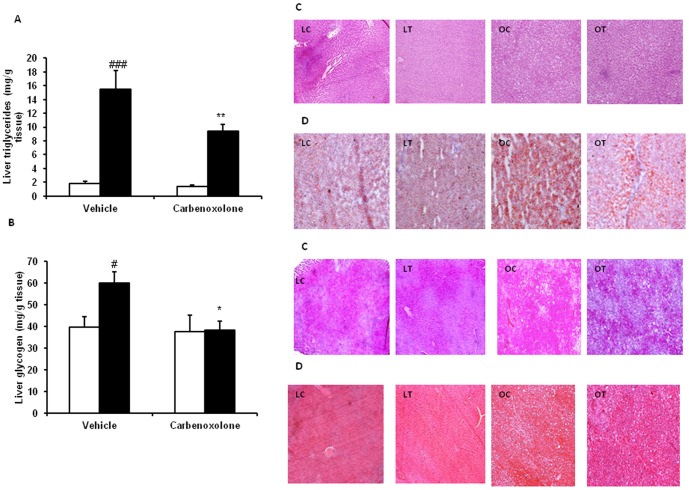
Effect of carbenoxolone on hepatic triglyceride and glycogen content. (A). Hepatic triglyceride content. (B). Hepatic glycogen content. (C). Photographs of liver tissue sections stained with haematoxylin and eosin (to study fat accumulation). (D). Photographs of liver tissue sections stained with oil Red O (to study fat accumulation). (E). Photographs of liver tissue sections stained with periodic acid-Schiff's stain (to study glycogen content). (F). Photographs of liver tissues sections stained with Masson's trichrome stain (to observe fibrosis). Parameters were measured after 4weeks of treatment with carbenoxolone or vehicle (50 mg/kg body weight/day). Empty bars indicate lean phenotype where as filled bars indicate obese phenotype. LC, Lean control; LT, Lean- treated; OC, Obese control; OT, Obese -treated. Images were taken at Magnification of 10X. Values are means ± SEM for 6 animals for group. #*p*<0.05, ##*p*<0.01 and ### *p*<0.001 comparing vehicle-treated lean and obese rats. *p<0.05, **p<0.01 and *** *p*<0.001 comparing carbenoxolone-treated animals with vehicle-treated animals of the same phenotype.

WNIN/Ob obese rats had significantly higher plasma triglycerides, total cholesterol and HDL-cholesterol as compared with lean rats (Figure 2) in both fasted and fed conditions. CBX treatment significantly decreased fed-state plasma triglycerides in obese rats (24%, p<0.05) but not in lean rats as compared with their respective vehicle-treated control rats (Figure 2). CBX brought down plasma triglycerides in obese rats approximately to that of vehicle-treated lean rats (Figure 3). Plasma triglyceride levels in fasted state were not affected by CBX in both the phenotypes (Figure 2).

CBX significantly decreased fasted and fed-state plasma total cholesterol levels in obese rats as compared with vehicle-treated control obese rats (36.5% and 31% respectively, p<0.001) (Figure 2). More importantly, after CBX treatment, both fasted and fed state- plasma total cholesterol levels in obese rats were comparable to those observed in control lean rats. However, CBX treatment had no impact on fasted or fed-state plasma total cholesterol levels of WNIN/Ob lean rats as compared with their respective untreated lean control rats (Figure 2).

CBX treatment significantly decreased both fasted and fed state plasma HDL-cholesterol levels in obese rats (p<0.001) as compared with those of vehicle-treated obese rats (Figure 2). In line with total cholesterol levels, HDL-cholesterol levels of CBX-treated obese rats also showed decline, which were comparable to those observed in control lean rats (Figure 2). CBX treatment had not altered the fasted or fed-state plasma HDL cholesterol levels in lean rats as compared with those of vehicle-treated lean rats (Figure 2).

### Effect of CBX on hepatic glycogen, triglycerides, steatosis and fibrosis

Vehicle-treated WNIN/Ob obese rat livers had significantly higher triglyceride (P<0.001) and glycogen (P<0.05) contents (Figure 3) as compared with those of vehicle-treated lean rats. CBX significantly reduced hepatic triglyceride (P<0.01) and glycogen contents (P<0.03) in obese rats but not in lean rats as compared with those of their respective control group rats (Figure 3). CBX significantly brought down obese rat- hepatic glycogen content to the level comparable to that of vehicle-treated lean rats. Histopathology showed decreased hepatic steatosis (H&E and Oil Red O staining) and glycogen (PAS staining) in livers of CBX-treated obese rats as compared with those of vehicle-treated obese rats (Figure 3). Hepatic fibrosis was not observed in any of the lean and obese rats of control and experimental groups (Figure 3).

### Effect of CBX on hepatic gene expression

**Figure 4:**
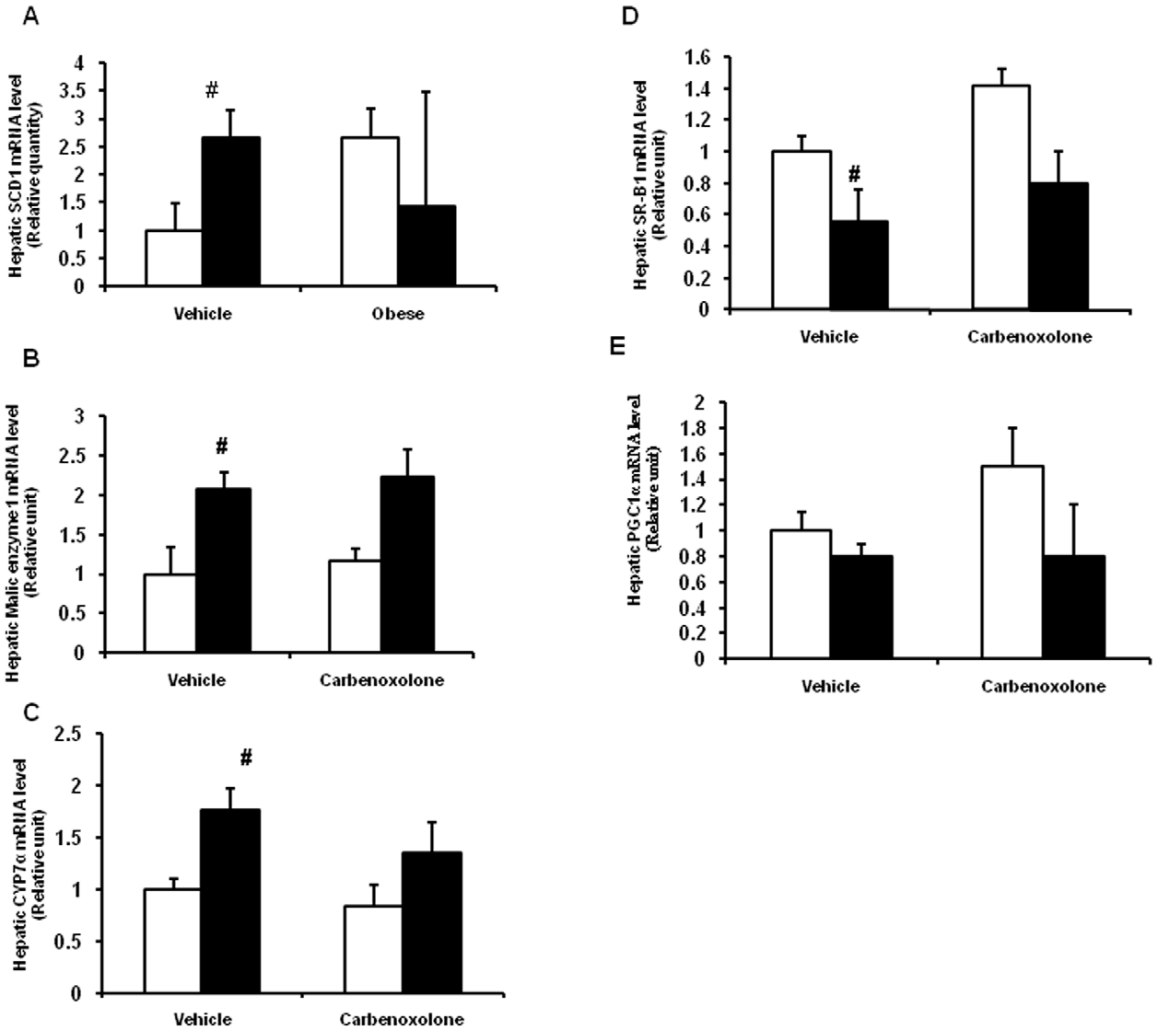
Effect of carbenoxolone on hepatic gene expression quantified by semi quantitative reverse transcription PCR. (A). Stearoyl CoA desaturase 1 (SCD1). (B). Malic enzyme 1 (ME1). (C). Cholesterol 7α-hydroxylase (CYP7α). (D). Scavenger receptor B1 (SR-B1). (E). PPARγ co-activator 1α (PGC1α). Relative gene expression was measured using glycerlaldehyde-3-phosphate dehydrogenase (GAPDH) gene as internal control. Relative expression of gene of interest in lean control was taken as 1. Parameters were measured after 4weeks of treatment with carbenoxolone or vehicle (50 mg/kg body weight/day). Empty bars indicate data from lean rats and filled bars indicate data from obese rats. Values are means ± SEM for 4 animals for group. . #*p*<0.05, ##*p*<0.01 and ### *p*<0.001 comparing vehicle-treated lean and obese rats. *p<0.05, **p<0.01 and *** *p*<0.001 comparing carbenoxolone-treated animals with vehicle-treated animals of the same phenotype.

We have studied the effect of CBX on the expression of genes involved in lipogenesis, fatty acid oxidation and cholesterol metabolism. The selection of genes was based on the observations from microarray analysis of liver in lean and obese rats (unpublished data and communicated else-where). Vehicle-treated obese rats have significantly higher expression of stearoyl CoA desaturase 1 (SCD1) (P = 0.03), malic enzyme 1 (ME1) (P = 0.02) and cholesterol-7α-hydroxylase (CYP7α) genes in liver as compared to those of vehicle-treated lean rats (Figure 4). Expression of SR-B1 (P<0.05) and PGC1α genes (P<0.05) were significantly lower in the liver of vehicle-treated obese rats as compared with those of vehicle-treated lean rats (Figure 4). CBX had not altered the expression any of the above-mentioned genes in both the phenotypes as compared with their respective control groups (Figure 4).

### Effect of CBX on food intake, body weight and tissue weights

CBX significantly reduced food intake in lean rats (p<0.01), but not in obese rats as compared with their respective vehicle-treated control rats (Table 1). There was no significant change in the body weight of CBX-treated lean and obese rats as compared with those of control group rats (Table 1). Body weight gain was significantly decreased by 29% in CBX-treated lean rats (p<0.05) but not in obese rats as compared with their respective, vehicle-treated control rats (Table 1). Among visceral adipose tissue depots, retroperitoneal and omental adipose tissue- weights showed a trend towards decrease in CBX treated- lean rats as compared with those of vehicle-treated lean rats (Table 1). On the other hand, in CBX-treated obese rats, omental and epididymal adipose depot weights showed a tendency towards decrease as compared to those of their respective control rats (Table 1). However, CBX treatment had not altered skeletal muscle mass in both the phenotypes (Table 1).

### Effect of CBX on body composition

**Figure 5:**
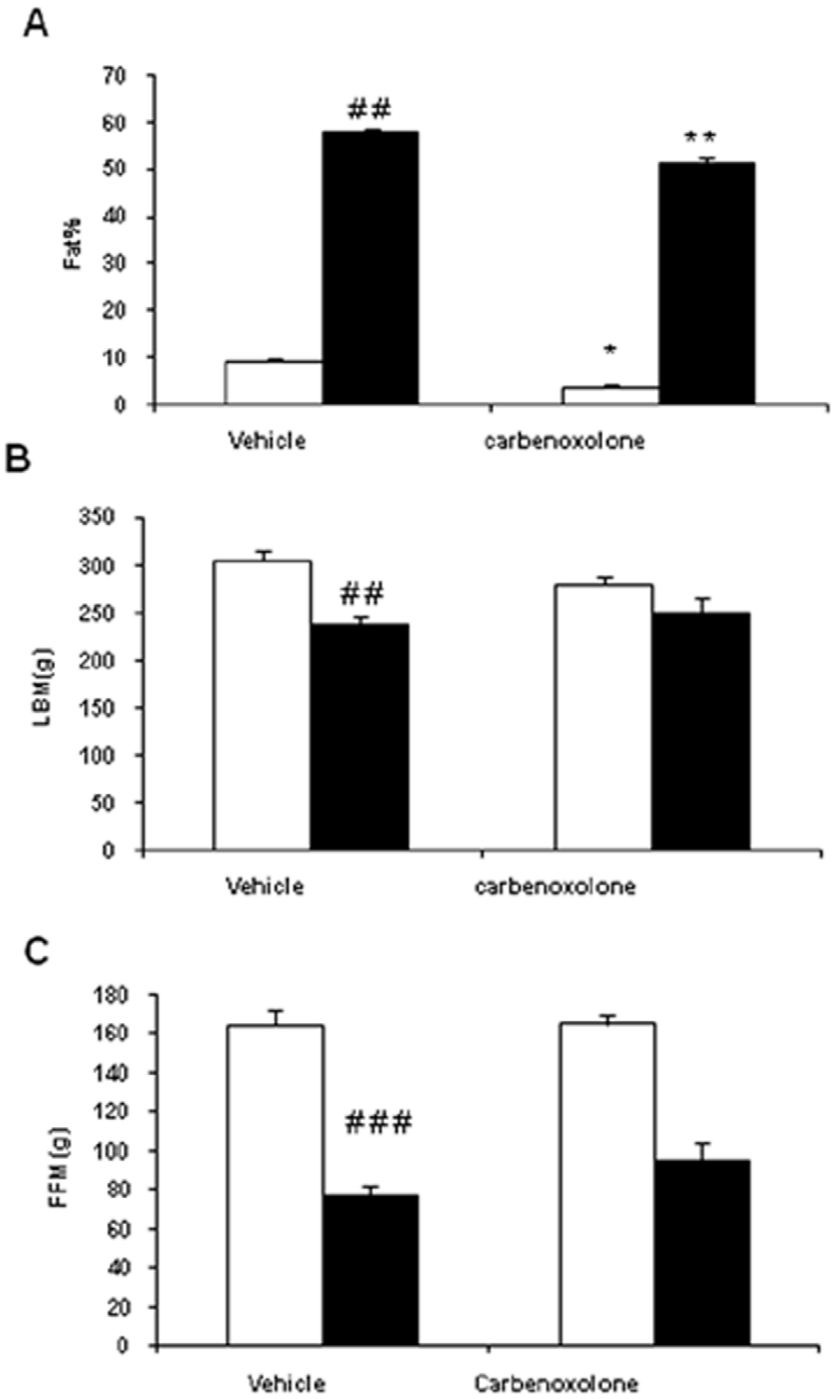
Effect of carbenoxolone on body composition of WNIN/Ob lean and obese rats. (A). fat percentage. (B). Lean body mass. (C). Fat free mass. Body composition was measured by TOBEC after 4weeks of treatment with carbenoxolone or vehicle (50 mg/kg body weight/day). Empty bars indicate lean phenotype where as filled bars indicate obese phenotype. Values are means ± SEM for 6 animals for group. #*p*<0.05, ##*p*<0.01 and ### *p*<0.001 comparing vehicle-treated lean and obese rats. *p<0.05, **p<0.01 and *** *p*<0.001 comparing carbenoxolone-treated animals with vehicle-treated animals of the same phenotype.

CBX treatment decreased fat percentage in lean (69%, p<0.01) and obese rats (13%, p<0.01) as compared with their respective vehicle-treated control rats (Figure 5). Lean body mass (LBM) was not altered by CBX treatment in WNIN/Ob lean and obese rats as compared with their respective control group rats (Figure 5). Fat-free mass (FFM) showed a strong tendency towards increase (p = 0.07) in CBX-treated obese rats as compared with that of vehicle- treated obese rats. However, no such changes were seen in CBX-treated lean rats (Figure 5).

### Effect of CBX on adipocyte hypertrophy, fibrosis and glycogen content

**Figure 6:**
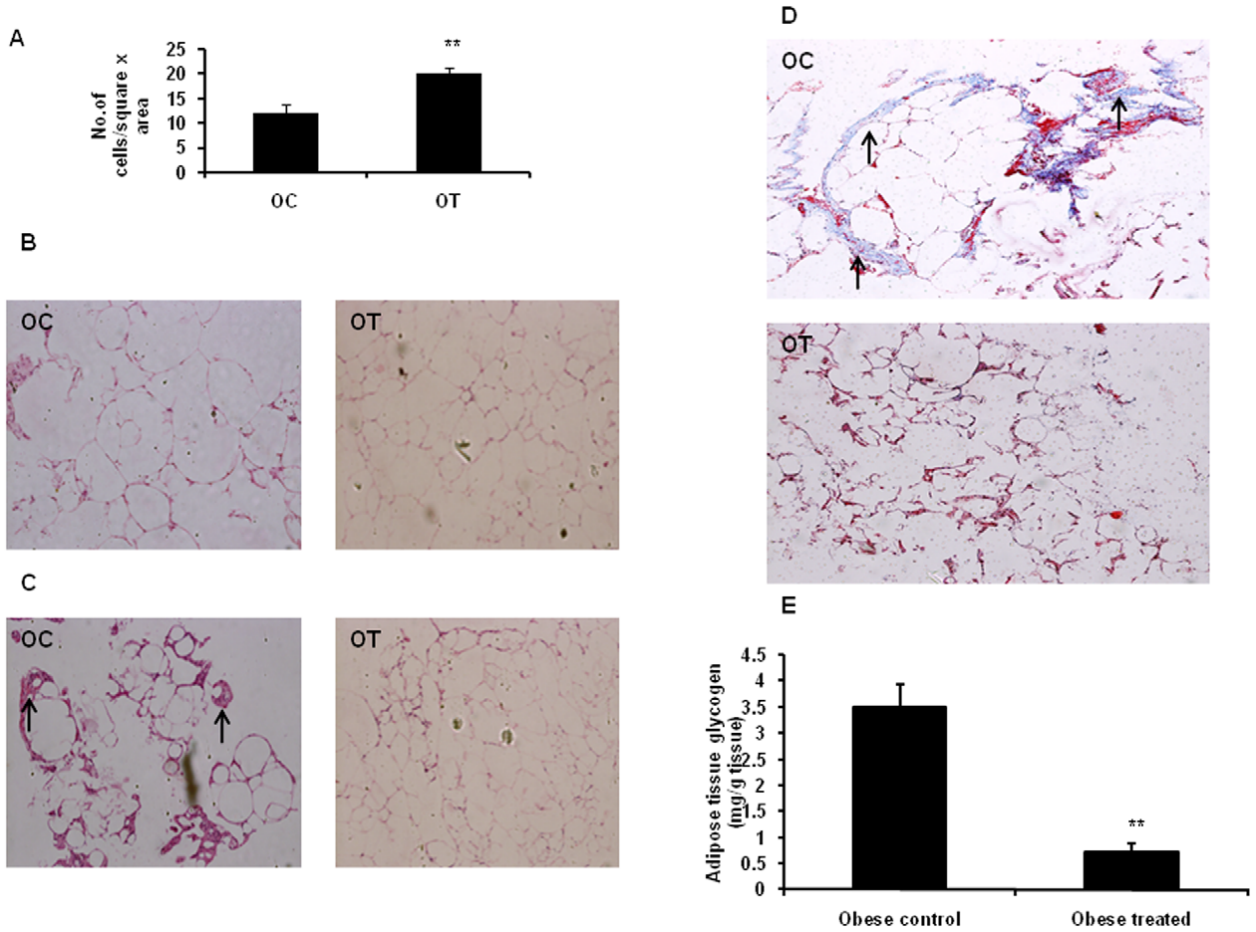
Effect of carbenoxolone on adipose tissue morphology and glycogen content. (A). Adipocyte size (number of cells/16sq.mm). (B). Photographs of adipose tissue sections stained with haematoxylin and eosin (to observe adipocyte cell size). (C). Photographs of adipose tissue sections with Haematoxylin and Eosin (to observe adipose tissue inflammation characterized by presence of ‘crown like structures’-indicated by arrows). (D). Photographs of adipose tissue sections stained with Masson's trichrome stain (to observe fibrosis-indicated by arrows). (E). Tissue glycogen content. Parameters were measured after 4weeks of treatment with carbenoxolone or vehicle (50 mg/kg body weight/day). Empty bars indicate lean phenotype, where as filled bars indicate obese phenotype. OC, Obese control; OT, Obese- treated. Images were taken at Magnification of 10X. Values are means ± SEM for 6 animals for group. . #*p*<0.05, ##*p*<0.01 and ### *p*<0.001 comparing vehicle-treated lean and obese rats. *p<0.05, **p<0.01 and *** *p*<0.001 comparing carbenoxolone-treated animals with vehicle-treated animals of the same phenotype.

Estimation of glycogen and histo-pathological studies were confined to the retroperitoneal adipose tissue of obese rats alone due to scarcity of adipose tissue in lean rats. Preliminary studies on adipose tissue of WNIN/Ob lean and obese rats showed increased adipocyte- hypertrophy, inflammation, fibrosis and glycogen content in obese rats (unpublished data). CBX decreased adipocyte hypertrophy and tissue fibrosis (Figure 6). Glycogen content in adipose tissue significantly decreased (P<0.01) by CBX treatment in adipose tissue of WNIN/Ob obese rats as compared with that of vehicle-treated obese rats (Figure 6). H&E staining revealed drastically decreased number of inflammatory cells in the extracellular spaces of adipose tissue specifically ‘crown-like structures’ (which are the hallmark of adipose tissue inflammation) in CBX – treated obese rats as compared with that of vehicle-treated obese rats (Figure 6).

### Effect of CBX on adipose tissue gene expression

**Figure 7:**
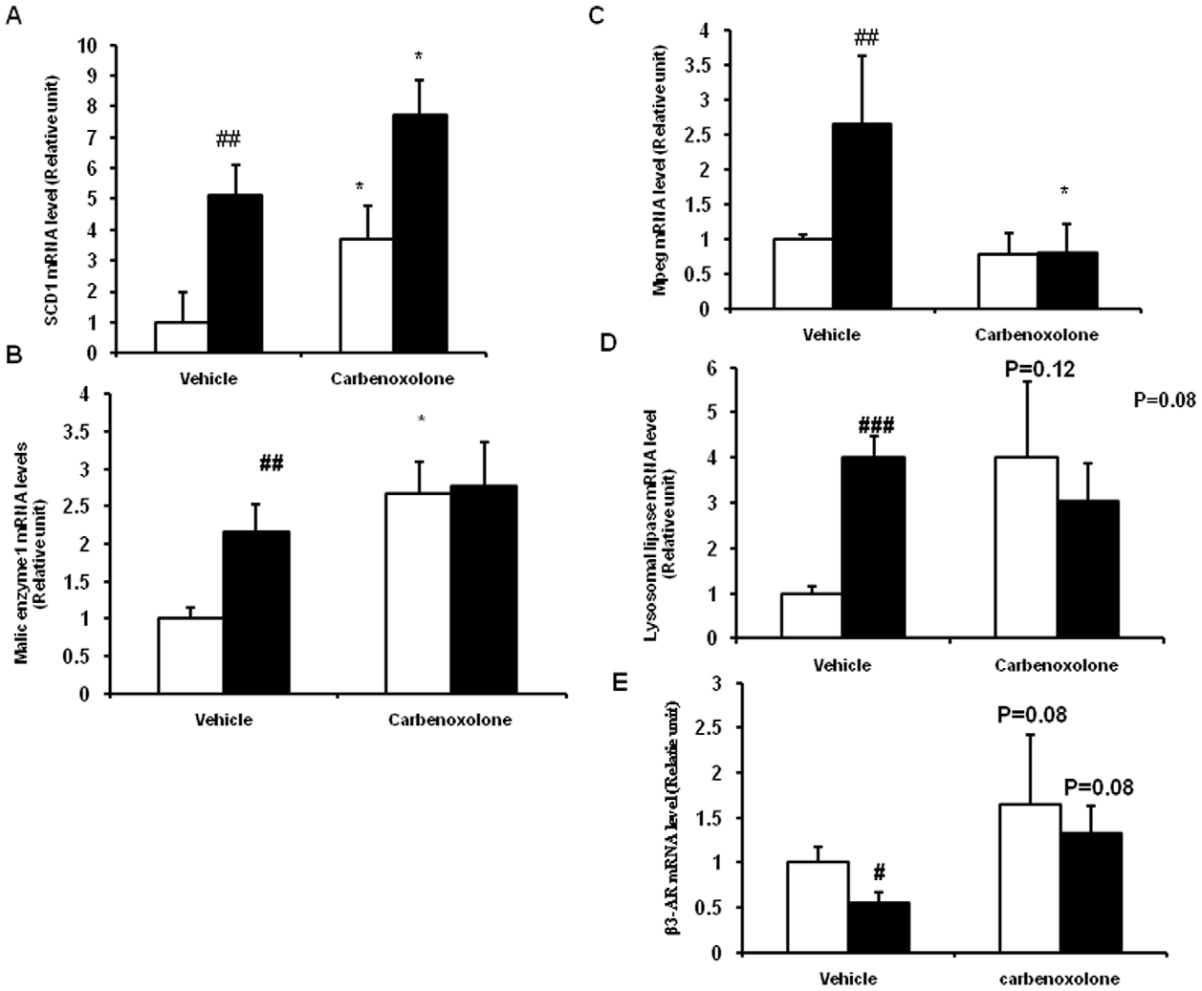
Effect of carbenoxolone on adipose tissue gene expression quantified by semi quantitative reverse transcription PCR. (A). Stearoyl CoA desaturase 1 (SCD1). (B). Malic enzyme 1 (ME1). (C). Macrophage expressed gene (MPEG). (D). Lysosomal acid lipase (LIPA). (E). Beta3-Adrenergic receptor (β3-AR). Parameters were measured after 4weeks of treatment with carbenoxolone or vehicle (50 mg/kg body weight/day). Empty bars indicate lean phenotype where as filled bars indicate obese phenotype. Relative gene expression was measured using calnexin gene as internal control. Relative expression of gene of interest in lean control was taken as 1. Values are means ± SEM for 4 animals for group. . #*p*<0.05, ##*p*<0.01 and ### *p*<0.001 comparing vehicle-treated lean and obese rats. *p<0.05, **p<0.01 and *** *p*<0.001 comparing carbenoxolone-treated animals with vehicle-treated animals of the same phenotype.

We have studied the effect of CBX on the expression of lipogenic genes and macrophage-specific genes in adipose tissue. The selection of genes was based on the observations from microarray analysis of adipose tissue in lean and obese rats (unpublished data). Vehicle-treated obese rats had significantly higher expression of SCD1 (P<0.01), ME1 (P<0.01), lysosomal acid lipase (LIPA) (P<0.01) and macrophage- expressed gene 1 (MPEG1) (P<0.05) in adipose tissue as compared with that of vehicle-treated lean rats (Figure 7). CBX significantly elevated the expression of SCD1 gene in the adipose tissue of both the phenotypes as compared with that of their respective vehicle-treated control groups (P<0.05) (Figure 7). ME1 gene expression was significantly increased by CBX in lean rats (P<0.05) but not in obese rats as compared with their respective control group rats (Figure 7). Tendency towards decrease in MPEG and LIPA gene expressions was observed in the adipose tissue of CBX-treated obese rats (P = 0.08) as compared with their respective control group rats (Figure 7). β3-adrenergic receptor (β3*-*AR) gene expression was significantly lower in adipose tissue of control obese rats as compared with that of control lean rats (P<0.01) (Figure 7). β3*-*AR gene expression in the adipose tissue of both the phenotypes (P = 0.08) showed a trend towards increase upon treatment with the CBX as compared with their respective control groups (Figure 7).

### Effect of CBX on insulin resistance, glucose- intolerance and insulin signaling

**Figure 8:**
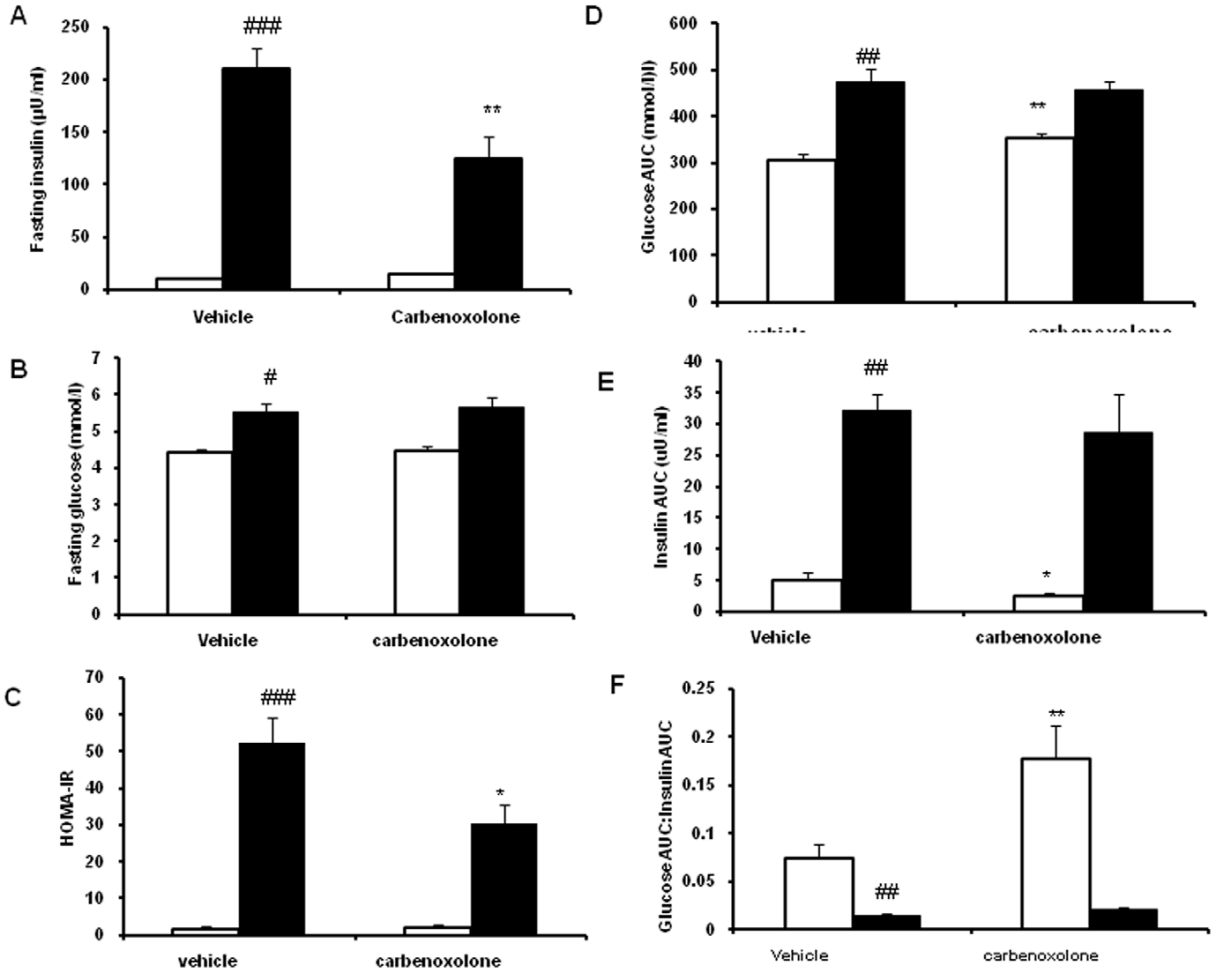
Effect of carbenoxolone on insulin sensitivity in WNIN/Ob lean and obese rats. (A). Fasting glucose. (B). Fasting insulin. (C). HOMA-IR. (D). Glucose AUC. (E). Insulin AUC. (F). Glucose AUC to Insulin AUC ratio. Parameters were measured after 4weeks of treatment with carbenoxolone or vehicle (50 mg/kg body weight/day). Empty bars indicate lean phenotype where as filled bars indicate obese phenotype. Values are means ± SEM for 6 animals for group. #*p*<0.05, ##*p*<0.01 and ### *p*<0.001 comparing vehicle-treated lean and obese rats. *p<0.05, **p<0.01 and *** *p*<0.001 comparing carbenoxolone-treated animals with vehicle-treated animals of the same phenotype.

CBX significantly decreased the fasting insulin in the obese rats (40%, p<0.01), but not in lean rats as compared with their respective vehicle-treated control group rats (Figure 8). CBX treatment had not altered fasting glucose levels in both the phenotypes as compared with their respective control groups (Figure 8). Insulin resistance as measured by HOMA-IR decreased significantly by CBX treatment in obese rats (p<0.05) but not in lean rats as compared with their respective vehicle-treated control group- rats (Figure 8).

**Figure 9:**
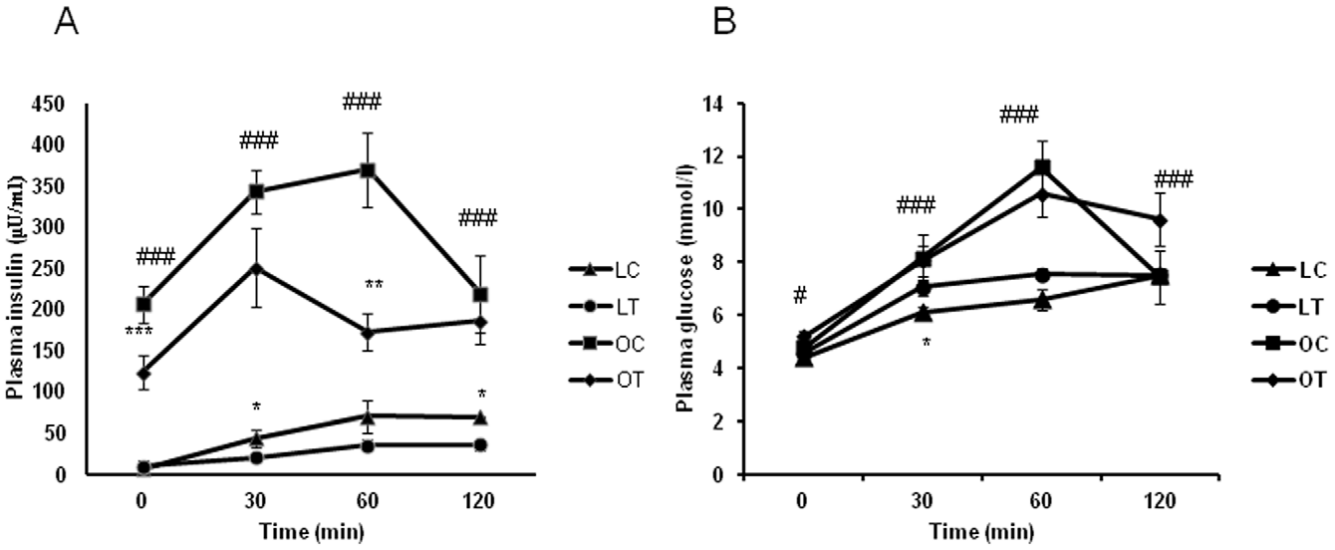
Effect of carbenoxolone on time course of plasma insulin (A) and glucose (B) during oral glucose tolerance test. Parameters were measured after 4weeks of treatment with carbenoxolone or vehicle (50 mg/kg body weight/day). Values are means ± SEM for 6 animals for group. #*p*<0.05, ##*p*<0.01 and ### *p*<0.001 comparing vehicle-treated lean and obese rats at the respective time point. *p<0.05, **p<0.01 and *** *p*<0.001 comparing carbenoxolone-treated animals with vehicle-treated animals of the same phenotype at the respective time point.

Glucose AUC, Insulin AUC and Glucose AUC to Insulin AUC were not altered by CBX in obese rats as compared with their respective vehicle-treated obese rats (Figure 8). Glucose AUC was significantly increased (p<0.01) and insulin AUC was significantly decreased (p<0.05) by CBX treatment in lean rats as compared with those of vehicle-treated lean rats (Figure 8). In CBX-treated lean rats, plasma insulin was significantly increased at 30 and 120 minutes after glucose challenge when compared with that of vehicle-treated lean rats. A strong trend (P = 0.08) towards increase in insulin level was also observed at 60 minutes in CBX-treated lean rats, when compared to that of control lean rats (Figure 9). On the contrary, plasma glucose level was significantly increased at 30 minutes after the glucose challenge in CBX-treated lean rats when compared to that of vehicle-treated lean rats (Figure 9). A strong trend towards increase (P = 0.10) in plasma glucose level was observed at 60 minutes after glucose challenge in CBX-treated lean rats as compared with that of control lean rats (Figure 9). Glucose to insulin AUC ratio was significantly increased in CBX-treated lean rats (p<0.01) as compared with those of vehicle-treated lean rats (Figure 8).

**Figure 10:**
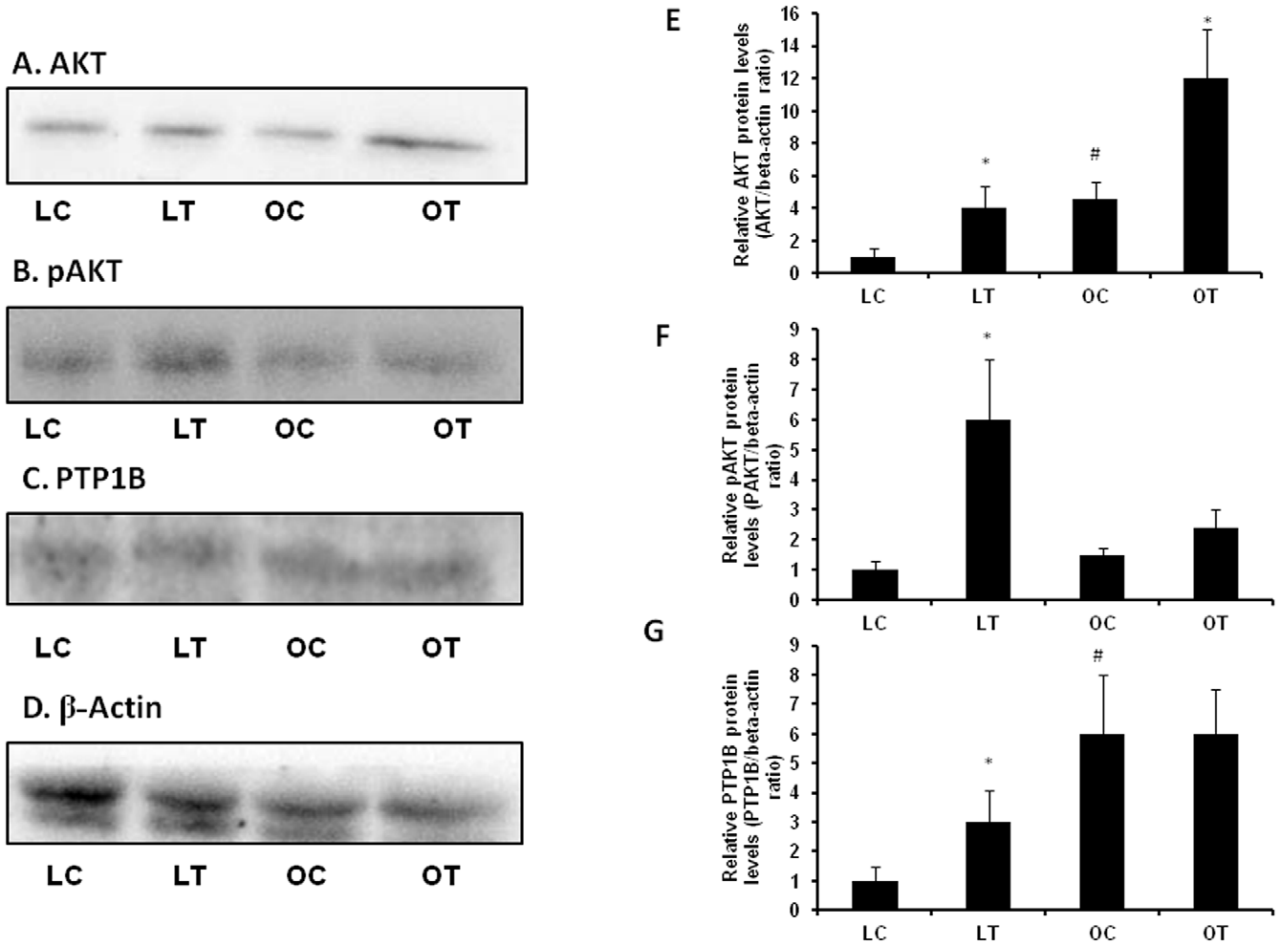
Effect of carbenoxolone on insulin signaling in gastrocnemius muscle of WNIN/Ob lean and obese rats. (A). Representative western blot for AKT. (B). Representative western blot for pAKT (Ser 473). (C). Representative western blot for PTP1B. (C). Representative western blot for β-actin. Parameters were measured after 4weeks of treatment with carbenoxolone or vehicle (50 mg/kg body weight/day). Relative protein levels were measured using β-actin as internal control. LC, Lean Control; LT, Lean Treated; OC, Obese Control; OT, Obese control. Relative expression of protein of interest in lean control was taken as 1. Values are means ± SEM for 4 animals for group. . #*p*<0.05 comparing vehicle-treated lean and obese rats. *p<0.05 comparing carbenoxolone-treated animals with vehicle-treated animals of the same phenotype.

To further understand the mechanisms involved in the differential effect of CBX on insulin sensitivity in WNIN/Ob lean and obese rats, we have studied the expression of insulin signaling proteins in muscle. Akt protein levels were significantly higher in the gastrocnemius muscle of obese rats as compared with that of lean rats (Figure 10). CBX-treatment significantly increased Akt protein levels in both the phenotypes (p<0.05) (Figure 10). pAkt levels were not significantly different between vehicle-treated lean and obese rats. CBX-treatment significantly increased pAkt levels in lean rats, but not in obese rats (p<0.05) (Figure 10). Expression of PTP1B protein was significantly higher in control obese rats as compared with those of control lean rats (p<0.05) (Figure 9). CBX-treatment significantly increased PTP1B levels in lean rats but not in obese rats (p<0.05) (Figure 10).

## Discussion

Here, we report the effect of 11β-HSD inhibitor CBX, on obesity and associated co-morbidities in WNIN/Ob obese rat, a novel genetically obese rat model. CBX treatment significantly inhibited 11β-HSD1 activity in the liver and adipose tissue of WNIN/Ob lean and obese rats. CBX treatment significantly lowered the elevated circulatory corticosterone levels of obese rats, suggesting amelioration of elevated hypothalamus-pituitary-adrenal axis (HPA axis) activity in obese rats. 11β-HSD1 inhibition decreased hypertriglyceridemia, hypercholesterolemia and hepatic steatosis in obese rats. 11β-HSD1 inhibition decreased body fat percentage, adipocyte- hypertrophy, ameliorated adipose tissue fibrosis in obese rats. Insulin sensitivity was improved by CBX treatment in obese rats. Interestingly, 11β-HSD1 inhibition induced severe fat loss and glucose- intolerance in lean rats. CBX corrected the elevated tissue glycogen contents of obese rat liver and adipose tissue .Thus, the results of our study suggest that 11β-HSD1 inhibition decreases obesity and ameliorates dyslipidemia and insulin resistance in WNIN/Ob obese rats. Further, our study supports the contention that inhibition of 11β-HSD1 can be a key strategy to treat metabolic syndrome. To our knowledge, this is the first study to relate 11β-HSD1 activity to adipose tissue fibrosis and tissue glycogen content in obese condition. This is also the first study to report the possible adverse effects of 11β-HSD1 inhibition in terms of fat loss and glucose- intolerance in lean rats.

### Hypothalamus-Pituitary-Adrenal (HPA) Axis

Plasma glucocorticoid levels are elevated in obese rodent models but in human obesity these are not altered or even low [23]. As an indication of elevated HPA axis activity, WNIN/Ob obese rats have heavier adrenal glands and elevated plasma corticosterone levels. 11β-HSD1 knock-out mice have elevated plasma corticosterone, where as transgenic mice with 11β-HSD1 over-expression in adipose tissue have normal plasma hormone levels [7], [8]. Further, 11β-HSD1 inhibition has not altered plasma hormone levels in human and animal models of obesity [23], [24]. In contrast to these observations, CBX significantly decreased plasma corticosterone levels in WNIN/Ob obese rats under fasting condition. Decreased hormone levels during fasting- state in obese rats by CBX could be due to the suppression of enhanced HPA axis activity under stress conditions (fasting).

### Circulatory TG, hepatic steatosis and gene expression

11β-HSD1 knock-out mice have lower plasma triglyceride levels, where as transgenic mice over-expressing 11β-HSD1 in adipose tissue or in liver have elevated plasma triglycerides [7], [8]. CBX and other selective 11β-HSD1 inhibitors have been shown to decrease the plasma triglyceride levels in different animal models of obesity and type 2 diabetic subjects [13]–[16]. CBX significantly decreased plasma triglyceride levels in WNIN/Ob obese rats in fed-state but not in fasting state. This observation is also reported in 11β-HSD1 knockout mice [8]. Further, 11β-HSD1 inhibition decreased triglyceride content in fatty livers of WNIN/Ob obese rats.

To understand the possible mechanisms involved in the amelioration of hypertriglyceridemia and hepatic steatosis by CBX, we studied the gene expression of lipogenic genes, SCD1 and ME1. SCD1 catalyses the conversion saturated fatty acids to monounsaturated fatty acids, which is essential for TG synthesis. ME1 provides the reducing equivalents (NADPH) for lipogenesis. Expressions of these lipogenic genes were elevated in the liver of WNIN/Ob obese rats. We also studied the gene expression of PGC1α, which acts as transcriptional co-activator for the expression of beta-oxidation genes. WNIN/Ob obese rats have lower expression of PGC1α in liver. Possibly, elevated lipogenic and lowered beta-oxidation genes might have contributed to hypertriglyceridemia and hepatic steatosis in WNIN/Ob obese rats. Lack of rescue of these dys-regulated genes by CBX in obese rats suggests the role of other mechanisms involved in CBX- mediated amelioration of hypertriglyceridemia and hepatic steatosis.

### Circulatory total cholesterol, HDL cholesterol and hepatic gene expression

In obese zucker rats, oral administration of CBX had not altered the plasma total cholesterol levels [10]. In contrast, subcutaneous injection of CBX to diet-induced obese mice and severely obese mice on LDL-receptor ^−/−^ background resulted in significant decrease in plasma total cholesterol levels [11]. On similar lines, subcutaneous administration of CBX to WNIN/Ob obese rats resulted in the amelioration of hypercholesteremia in both fed and fasted state and brought down the levels to those observed in control lean rats.

In majority of the obese rodent models, plasma HDL-cholesterol levels are not altered or even low despite elevated total cholesterol [25]. Conversely in leptin- deficient *ob*/*ob* mice and leptin- resistant *db*/*db* mice, elevated plasma cholesterol levels are associated with significantly higher HDL-cholesterol levels [25]. Similar to that of *ob/ob* and *db/db* mice, WNIN/Ob obese rats have elevated HDL-cholesterol levels. Mechanisms relating 11β-HSD1 activity and plasma HDL-cholesterol levels are not clearly understood. 11β-HSD1 knockout-mice have elevated plasma cholesterol levels with significantly higher HDL-cholesterol, on the contrary, transgenic mice over-expressing hepatic 11β-HSD1 have unaltered plasma total and HDL-cholesterol levels [7], [8]. CBX significantly decreased fasted and fed-state plasma HDL-cholesterol levels in WNIN/Ob obese rats.

One of the mechanisms involved in the elevation of plasma HDL-cholesterol in this model was the down-regulation of hepatic scavenger receptor B1 (SR-B1) [26]. SR-B1 mediates the selective uptake of cholesterol esters from plasma HDL-particles, thus plays crucial role in reverse cholesterol transport [26]. To explain the possible mechanism involved in CBX-mediated decrease in HDL-cholesterol, we have studied the hepatic SR-B1 gene expression. 11β-HSD1inhibition by CBX did not correct the observed under-expression of hepatic SR-B1 in obese rats, indicating the involvement of other mechanisms in lowering of plasma cholesterol. To understand the mechanism involved in lowering of plasma total and HDL-cholesterol levels, we studied the expression of CYP7α gene, which catalyses the rate limiting step in bile acid synthesis. CYP7α gene expression was higher in obese rats, suggesting elevated bile acid synthesis, which may be a compensatory mechanism to normalise the elevated plasma cholesterol in obesity. 11β-HSD1 inhibition did not alter the hepatic CYP7α gene expression in obese rats. Previous studies have shown that 11β-HSD1inhibition decreases the expression of genes involved in cholesterol biosynthesis [11]. As genes involved in cholesterol excretion (CYP7α) or HDL uptake (SR-B1) were not affected by 11β-HSD1 inhibition in WNIN/Ob obese rats, possibly, decreased expression of cholesterol biosynthetic genes might have contributed to the amelioration of hypercholesterolemia in this model. Although CBX decreased plasma HDL cholesterol in obese rats, it may not be pro-atherogenic, as the levels were brought down to those observed in lean rats. Thus, the decreased plasma HDL-cholesterol level in obese rats is the consequence of cholesterol removal from body. This phenomenon is considered to be anti-atherogenic.

### Body composition, adipose tissue morphology, fibrosis and gene expression

Previous studies have reported that cortisol (corticosterone in rodents) is essential for preadipocyte differentiation [27]. 11β-HSD1 plays an important role in adipose tissue physiology, as it increases local corticosterone levels. Adipose-specific over- expression of 11β-HSD1 in mice, results in increased visceral fat mass due to adipocyte- hypertrophy, where as 11β-HSD1 knock-out mice are resistant to diet-induced obesity [7], [8]. Elevated 11β-HSD1 activity was observed in adipose tissue of obese rodent models and in human obesity [4]–[6].11β-HSD1 inhibition by CBX or selective inhibitors resulted in decreased fat mass in diet-induced obesity and rodent models of genetic obesity [11], [16]. In line with these observations, 11β-HSD1 inhibition significantly decreased body fat mass in WNIN/Ob lean and obese rats. As observed in previous studies [28], 11β-HSD1 inhibition decreased adipocyte- hypertrophy in obese rats.

To further explain the molecular mechanisms involved the adipose tissue loss, we have studied the expression of beta3-adrenergic receptor (β3-AR) gene, which stimulates lipolysis in adipose tissue. β3-AR gene expression is shown to be lower in obese rodent models and its activation leads to fat loss and amelioration of obesity- induced insulin resistance [29], [30]. Recent studies on 11β-HSD1 knock-out mice also showed increased beta-adrenergic signaling in visceral adipose tissue [31]. In line with the previous observations, WNIN/Ob obese rats have reduced β3-AR gene expression in adipose tissue. An increased trend in β3-AR gene expression was also observed in adipose tissue of CBX-treated obese rats, which could be one of the mechanisms involved in fat loss observed in the obese rats.

Notably, CBX induced severe fat- loss in lean rats (69%) as compared with that observed (13%) in obese rats. This might be due to the fact that 11β-HSD1 inhibition by CBX was higher in adipose depots of lean rats than that of obese rats. Another possible reason for the observed excessive fat- loss in lean rats might be due to decreased food intake by CBX administration, which was not affected in obese rats. As in case of obese rats, a trend towards increase was also observed in adipose tissue- β3-AR gene expression of CBX-treated lean rats. Elevated β3-AR expression by CBX might have resulted in lipolysis and fat mobilization in lean rats. To our knowledge, this is the first study to show the effect of 11β-HSD1 inhibition on adipose tissue β3-AR gene expression.

Recent studies have shown that extracellular matrix plays an important role in adipose tissue physiology. Adipose tissue fibrosis is reported in obese animal models and humans [32]. Amelioration of obesity and insulin resistance is known to decrease adipose tissue fibrosis in animal models of obesity [32]. So far, no studies have reported the effect of 11β-HSD1 inhibition on adipose tissue fibrosis in obese and insulin- resistant conditions. 11β-HSD1 inhibition ameliorated fibrosis in adipose tissue of WNIN/Ob obese rats. Previous studies have reported that improvement in insulin-sensitivity decreases adipose tissue fibrosis by decreasing the expression of collagen genes [32]. Possibly, the observed improvement in insulin-sensitivity by 11β-HSD1 inhibition might have decreased the adipose tissue fibrosis by down-regulating collagen genes in this model.

### Insulin resistance and Glucose- intolerance

11β-HSD1 knock-out mice have improved insulin sensitivity, where as the transgenic over-expression of 11β-HSD1 in liver or adipose tissue results in the development of insulin resistance [7]–[9]. Oral administration of CBX has increased the fasting insulin levels without affecting glucose- intolerance in obese zucker rats [10]. In contrast to this, subcutaneous administration of CBX has resulted in decreased fasting insulin levels in severely-obese mice on LDLR^−/−^ background [23]. Selective inhibition of 11β-HSD1 has resulted in decreased hyperglycemia and hyperinsulinaemia in *ob/ob*, *db/db* mice, but not altered glucose intolerance [13,14, and 16]. In line with the results of the previous studies, CBX treatment decreased hyperinsulinaemia in WNIN/Ob obese rats, suggesting improved peripheral insulin sensitivity.

Along with decreased plasma and tissue corticosterone levels, decreased fat mass might have improved insulin- sensitivity in obese rats. Another possible mechanism involved in the improved insulin sensitivity in obese rats might be due to decreased adipose tissue inflammation. Increased infiltration of immune cells like macrophages into adipose tissue was observed in human and animal obesity and was reported to diminish local and systemic insulin sensitivity [33]. Histology studies showed decreased number of immune cells in extracellular spaces around the adipocytes in CBX-treated obese rats. “Crown like structures” in adipose tissue, which are reported to be the hall mark of adipose tissue inflammation were decreased in adipose tissue of CBX-treated obese rats [33]. In support to this, gene expression studies showed a strong tendency towards decreased expression of macrophage- expressed gene 1 (MPEG1) and lysosomal acid lipase (LIPA) genes in adipose tissue of CBX-treated obese rats. MPEG1 is one of the marker genes for macrophages and the expression of LIPA gene in adipose tissue is shown to be mainly contributed from stromal vascular fraction, especially, from immune cells like macrophages [34]. Protein kinase B/Akt is a key component of insulin signaling and is shown to increase insulin sensitivity by enhancing GLUT4 translocation [35]. Protein-tyrosine kinase 1B (PTP1B) is a negative regulator of insulin signaling and is known to play critical role in insulin resistance [36]. 11β-HSD1 inhibition neither increased the activated Akt (phosphorylated- Akt) levels in muscle of obese rats and nor corrected the elevated PTP1B levels, suggesting that the improved insulin sensitivity could be mainly due to decreased adipose tissue inflammation not due to improved insulin signalling mechanisms in skeletal muscle.

In contrast to the observation in obese rats, CBX treatment induced glucose intolerance in lean rats. This is possibly due to decreased secretion of insulin from the pancreas upon glucose challenge as insulin levels were not elevated as compared to control lean rats after oral glucose load at all time points. Along with the decreased insulin release from pancreas, loss of significant amount of fat mass might have also contributed to the glucose- intolerance in lean rats (adipose tissue being one of the main organs for insulin- stimulated glucose uptake). Thus, CBX treatment (11β-HSD1 inhibition) caused glucose-intolerance in lean rats. Interestingly, it also increased AKT and pAKT protein levels along with PTP1B in skeletal muscle. This could be a compensatory mechanism, (which helps in enhancing muscle glucose uptake through elevated AKT & pAKT) to offset the elevated glucose levels arising out of decreased plasma insulin levels and excessive fat loss. On the other hand, increased PTP1B levels help in attenuating enhanced-insulin signaling.

### Tissue glycogen content

11β-HSD1 KO mice have ten-fold higher hepatic glycogen than normal mice, indicating that 11β-HSD1 plays significant role in glycogen metabolism [8]. In human and animal obesity, glycogen levels are elevated in liver [37], [38]. The exact mechanisms involved in abnormal glycogen metabolism in obesity are not clearly understood. Elevated hepatic and adipose tissue glycogen in WNIN/Ob obese rats suggest altered glycogen metabolism as observed in animal and human obesity. 11β-HSD1 inhibition by CBX decreased the elevated glycogen content in the liver and adipose tissue of WNIN/Ob obese rats, providing evidence that 11β-HSD1 inhibition can correct dys-regulated glycogen metabolism associated with obesity. Lowering of elevated circulatory and hepatic triglycerides along with hepatic and adipose tissue glycogen contents by CBX, indicates that 11β-HSD1 inhibition can correct the impaired fuel metabolism and insulin resistance, the hall marks of obesity. To our knowledge, this is the first study to report that inhibition of 11β-HSD1 decreases glycogen contents of liver and adipose tissue, a phenomenon associated with obesity and insulin-resistance.

### Conclusion

In summary, these data suggest that 11β-HSD1 inhibition by CBX decreases fat percentage, adipocyte-hypertrophy and ameliorates metabolic abnormalities like hypertriglyceridemia, hypercholesteremia and insulin resistance in WNIN/Ob obese rats. 11β-HSD1 inhibition ameliorates obesity-associated adipose tissue fibrosis, inflammation and also corrected the elevated hepatic and adipose tissue glycogen in WNIN/Ob obese rats. Finally, we conclude that 11β-HSD1 inhibition by carbenoxolone decreases obesity and ameliorated co-morbidities like dyslipidemia, insulin resistance in WNIN/Ob obese rat, a novel rat model for genetic obesity. Undoubtedly, the outcome of this study strongly supports the contention that 11β-HSD1 inhibition is a key strategy to ameliorate metabolic abnormalities associated with obesity. However, arguably, the observations in lean rats especially, severe fat- loss and glucose -intolerance by 11β-HSD1 inhibition, caution us against extending this strategy to insulin resistant-normal (lean) individuals.
